# Full-Fat Black Soldier Fly Larvae Meal in Diets for Pacu, *Piaractus brachypomus*: Digestibility, Growth Performance, Blood Biochemistry and Gut Morphology †

**DOI:** 10.3390/ani16172741

**Published:** 2026-09-02

**Authors:** Ruben Cuadros-Cuya, Victor Jesús Vergara-Rubín, Ignacio Jauralde-Garcia

**Affiliations:** 1 Department of Nutrition, Faculty of Animal Science, Universidad Nacional Agraria La Molina, Lima 15024, Peru; 2 Programa de Investigación y Proyección Social en Alimentos (PIPSA), Department of Nutrition, Universidad Nacional Agraria La Molina, Lima 15024, Peru; vjvergara@lamolina.edu.pe; 3 Laboratorio de Investigación en Nutrición y Alimentación en Peces y Crustáceos (LINAPC), Department of Nutrition, Universidad Nacional Agraria La Molina, Lima 15024, Peru; 4 Aquaculture and Biodiversity Research Group, Institute of Animal Science and Technology, Universitat Politècnica de València, Camino de Vera 14, 46071 València, Spain

**Keywords:** *Piaractus brachypomus*, *Hermetia illucens*, insect meal, full-fat black soldier fly, aquafeed, fishmeal replacement, digestibility, gut morphology

## Abstract

Black soldier fly larvae meal has emerged as a sustainable alternative to fishmeal (FM) in aquafeeds. However, the use of full-fat black soldier fly larvae meal (FF-BSFL) may be limited by its relatively high lipid and chitin contents, which could affect nutrient digestibility and fish performance. This study determined the apparent digestibility coefficients (ADCs) of FF-BSFL and evaluated the effects of replacing 0%, 30%, 60%, and 100% of FM with FF-BSFL in diets for juvenile pacu (*Piaractus brachypomus*). A 60-day feeding trial was conducted using 320 juvenile pacu (initial weight: 3.0 g) to evaluate growth performance, serum biochemical parameters, and intestinal histomorphology. The results showed high ADCs for protein (88.76%), lipids (97.09%), and gross energy (85.96%) in FF-BSFL. Fish fed the diet containing 30% FM replacement exhibited growth performance comparable to the control group. However, replacement levels of 60% and 100% significantly reduced growth and feed efficiency. Furthermore, high fishmeal replacement levels decreased mucosal thickness and muscularis thickness, reduced goblet cell abundance, and increased serum triglyceride concentrations and aspartate aminotransferase (AST) activity. In conclusion, FF-BSFL was highly digestible for juvenile pacu. Replacement of up to 30% of FM maintained growth performance and intestinal integrity, whereas higher replacement levels negatively affected growth performance and intestinal health.

## Simple Summary

Fishmeal is an important ingredient in aquafeeds, but its cost and limited sustainability have increased interest in sustainable alternatives. This study evaluated full-fat black soldier fly larvae meal as a replacement for fishmeal in diets for juvenile pacu (*Piaractus brachypomus*). The ingredient showed high digestibility, and replacing 30% of fishmeal maintained growth performance, blood biochemical status, and intestinal integrity. However, higher replacement levels reduced feed intake and growth and affected intestinal health. These results suggest that full-fat black soldier fly larvae meal can be used as a partial replacement for fishmeal in juvenile pacu diets.

## 1. Introduction

Aquaculture is the fastest-growing food production sector globally and plays a crucial role in mitigating food insecurity and malnutrition [1,2]. Traditionally, fishmeal (FM) and fish oil have been the main sources of essential proteins and lipids in aquaculture feed formulations due to their excellent amino acid profile, high digestibility, and palatability [3,4]. However, the overexploitation of pelagic fishery resources, the constant increase in their price, competition with other animal production sectors, and remote sourcing for non-South American countries have turned fishmeal and fish oil into ecologically and economically unsustainable resources in the long term, driving the search for alternative, cost-effective, and sustainable protein and lipid sources [5,6].

In this context, insect meals have emerged as one of the most promising and sustainable options. In particular, the black soldier fly (*Hermetia illucens*) stands out for its remarkable ability to efficiently biotransform organic waste into biomass with high nutritional value, requiring minimal water and agricultural land use, and generating a low environmental carbon footprint [7,8]. Black soldier fly larvae meal (BSFL) has a high protein (40–60%) and lipid (10–35%) content, as well as an essential amino acid profile comparable to that of fishmeal [9,10]. Furthermore, these larvae are a natural source of bioactive compounds, such as lauric acid and chitin, which have demonstrated antimicrobial, prebiotic, and immunostimulatory properties capable of promoting gut health and disease resistance in fish [11,12]. However, undesirable effects have also been reported, such as decreased protein digestibility or loss of feed palatability due to excessive lauric acid [13,14].

Most previous studies have focused on the use of defatted black soldier fly larvae meal (DF-BSFL) to minimize lipid oxidation, increase crude protein content, and facilitate the formulation of diets with standardized energy levels [8,15]. However, the use of full-fat black soldier fly larvae meal (FF-BSFL) may be economically advantageous because it eliminates the costly defatting process [16]. Furthermore, some of the most commonly used defatting methods rely on organic solvents, which reduce the sustainability advantages associated with this ingredient [17].

Because full-fat black soldier fly larvae meal contains higher levels of lipids and chitin, which are inherent components of the insect exoskeleton, high dietary inclusion levels may reduce digestibility and impair nutrient utilization and absorption [18,19]. However, many omnivorous fish species show good tolerance to chitin and possess gastric chitinase activity that facilitates its utilization [11].

The pacu (*Piaractus brachypomus*) is an omnivorous fish native to the Amazon basin that has high commercial value and characteristics ideal for aquaculture, such as rapid growth and great adaptability. While it has been previously demonstrated that the inclusion of defatted BSFL meal in phylogenetically close species, such as the tambaqui (*Colossoma macropomum*), allows for the replacement of some fishmeal without harming growth and even improving it at controlled levels [20], information on the physiological and zootechnical viability of using high levels of FF-BSFL meal specifically in pacu diets remains scarce.

Therefore, the purpose of this study was to evaluate the apparent digestibility of full-fat black soldier fly larvae meal (FF-BSFL) and its effectiveness as a substitute for fishmeal (FM) in diets for juvenile pacu, analyzing its effects on growth performance, blood biochemistry, and intestinal histomorphology. In this context, the aim is to determine the optimal level of fishmeal replacement with FF-BSFL that allows for adequate growth and physiological well-being in this species.

## 2. Materials and Methods

### 2.1. Location and Experimental Period

The study was conducted in two experimental phases. The digestibility trial was carried out at the Laboratory for Research on Fish and Crustacean Nutrition and Feeding (LINAPC), Department of Nutrition, Faculty of Animal Science, Universidad Nacional Agraria La Molina (UNALM), during April and May 2025. The experimental diets were manufactured at the Animal Feed Plant of the Food Research and Social Outreach Program (PIPSA), Faculty of Animal Science, (UNALM).

The growth trial was conducted at the facilities of the aquaculture company Pucayagro, located in the district of Calzada, province of Moyobamba, department of San Martín, Peru, between June and August 2025. The study was approved by the Ethics Committee of Universidad Nacional Agraria La Molina (UNALM) under protocol No. 20-2025-CEI-UNALM.

### 2.2. Biological Material

For the digestibility trial, nine pacu (*Piaractus brachypomus*) were obtained from the Fisheries Station of Universidad Nacional Agraria La Molina (Lima, Peru). Fish were randomly distributed and had an average body weight of 454.5 ± 47.81 g and an average total length of 26.93 ± 0.97 cm. For the growth trial, 320 fingerlings, approximately 45 days old, were obtained from Ambioterra (San Martín, Peru), with an initial average body weight of 3.0 g and an average total length of 5.5 cm.

### 2.3. Hermetia illucens Larvae Meal

Full-fat black soldier fly (*Hermetia illucens*) larvae meal was obtained from a commercial supplier (BSF Logistics, Lima, Peru). Larvae at the prepupal stage, selected because this stage is characterized by the highest accumulation of protein, lipid, and bioactive compound reserves prior to metamorphosis, were thermally dehydrated (140 °C for 20 min) and subsequently ground to an approximate particle size of 300 μm.

The meal was characterized by proximate analysis, including moisture, crude protein, ether extract, crude fiber, and ash, according to the official methods of the Association of Official Analytical Chemists [21]. Gross energy was determined using an oxygen bomb calorimeter (Model 1341EE, Parr Instrument Company, Moline, IL, USA) following ASTM method D-2015-66 (1974) [22]. The amino acid profile was analyzed by ultra-performance liquid chromatography with diode-array detection, using an ACQUITY UPLC H-Class PLUS system (Waters Corporation, Milford, MA, USA), whereas the fatty acid profile was determined by gas chromatography (Agilent 7890B; Agilent Technologies, Santa Clara, CA, USA). Heavy metals (mercury, cadmium, and lead) were quantified using an Agilent 5800 ICP-OES system (Agilent Technologies, Santa Clara, CA, USA) and a PinAAcle 900H atomic absorption spectrometer (PerkinElmer Inc., Waltham, MA, USA), and all values were below the maximum permissible limits. Microbiological analyses for *Salmonella* spp. and *Escherichia coli* were also performed, yielding negative results.

### 2.4. Digestibility Trial

#### 2.4.1. Experimental Design and Diets of the Digestibility Trial

The digestibility trial was conducted using a completely randomized design with nine Guelph-type digestibility tanks integrated into a recirculating aquaculture system (RAS). Each tank was equipped with a fecal collection column at the bottom. One fish was stocked per experimental unit, and three replicates were assigned to each dietary treatment.

A reference diet was formulated and supplemented with 0.5% chromium oxide (Cr_2_O_3_) as an inert marker. Two test diets were prepared to determine the apparent digestibility coefficients (ADCs) of fishmeal (FM) and full-fat black soldier fly larvae meal (FF-BSFL). Each test diet consisted of 69.5% of the reference diet, 30% of the test ingredient, and 0.5% Cr_2_O_3_. All diets were formulated to contain 34% crude protein, according to the requirement recommended for pacu by Briones [23].

Ingredients were ground using a disc mill (average particle size of 100 μm), weighed, mixed, and extruded using a twin-screw extruder (DRX-300 H Turbo Extruder, Food and Machines, Ribeirão Preto, SP, Brazil). Representative samples of the diets were subsequently analyzed for proximate composition (Table 1).

#### 2.4.2. Feeding and Fecal Collection

During the two-week acclimation period, fish were gradually adapted to their assigned experimental diets, but feces were not collected during this period. After the acclimation period, fish were fed to apparent satiation twice daily (08:00 and 18:00 h). Feces were collected using the sedimentation method twice daily before feeding. The fecal collection period lasted 37 days. Collected feces were dried at 65 °C, ground, and stored at −4 °C until chemical analysis.

#### 2.4.3. Calculation of Apparent Digestibility Coefficient

The apparent digestibility coefficient (ADC) of dry matter, nutrients, and gross energy were calculated according to the equations proposed by National Research Council [24], based on the ratio between the inert marker concentrations in the diet and feces:

ADC (%) = 100 − [100 × ((%Cr_2_O_3_ in diet)/(%Cr_2_O_3_ in feces)) × ((% nutrient (or energy) in feces)/(% nutrient (or energy) in diet))]

The ADC of the test ingredients were subsequently calculated using the following equation:

ADCi (%) = ADCtd + (ADCtd − ADCref) × ((r × Nref)/(i × Ni))

where: ADCi is the apparent digestibility coefficient of the ingredient; ADCtd is the apparent digestibility coefficient of the nutrient in the test diet; ADCref is the apparent digestibility coefficient of the nutrient in the reference diet; r is the proportion of the reference diet in the test diet (0.695); i is the proportion of the test ingredient in the test diet (0.30); Nref is the nutrient concentration in the reference diet (%); and Ni is the nutrient concentration in the test ingredient (%).

### 2.5. Growth Trial

#### 2.5.1. Experimental Design and Diets of the Growth Trial

The growth trial was conducted using a completely randomized design with four treatments and four replicates (16 tanks of 150 L; 20 fish per tank). The experimental units were part of a recirculating aquaculture system (RAS) with constant aeration and a natural photoperiod. Four isonitrogenous and isoenergetic diets were formulated: a control diet based on fishmeal and three diets containing increasing levels of full-fat black soldier fly larvae meal (FF-BSFL), replacing 30%, 60%, and 100% of the fishmeal. These treatments were designated as HI0, HI30, HI60, and HI100, respectively. The formulation and calculated nutrient composition of the experimental diets are presented in (Table 2). All ingredients were ground, sieved (0.8 mm), and extruded using a twin-screw extruder (DRX-300 H Turbo Extruder, Food and Machines, Ribeirão Preto, SP, Brazil), producing pellets of 0.5, 1.0, 1.5, 2.0 and 3.0 mm. Representative samples of the diets were analyzed to determine their proximate composition (Table 3).

#### 2.5.2. Management and Feeding

Fish were fed to apparent satiation throughout the experimental period. During the first two weeks, fish were fed six times daily; during weeks 3 and 4, feeding frequency was reduced to five times daily; during weeks 5 and 6, to four times daily; and thereafter to three times daily until the end of the trial. The feeding trial lasted 60 days.

#### 2.5.3. Growth Performance Parameters and Sampling

Fish were sampled at the beginning of the experiment and on days 15, 30, 45, and 60. During intermediate biometric samplings, fish were anesthetized with eugenol (30 mg L^−1^), weighed, measured, and returned to their respective tanks after recovery from anesthesia. The following growth performance parameters were calculated:

- Weight gain (g) = final weight − initial weight;
- Final biomass (g) = final number of fish × final average weight;
- Feed intake (g) = total feed consumed per tank;
- Feed conversion ratio (FCR) = feed intake/biomass weight gain;
- Specific growth rate (SGR, % day^−1^) = 100 × [(ln final weight − ln initial weight)/days];
- Condition factor (CF) = 100 × (body weight/total length^3^);
- Protein efficiency ratio (PER) = biomass weight gain/protein intake.

### 2.6. Water Quality Parameters

During the digestibility and growth trials, water quality parameters were monitored to ensure suitable conditions for the species. Temperature, pH, and dissolved oxygen were recorded daily, whereas water hardness, total ammonia nitrogen, nitrite, and nitrate were measured three times per week (Table 4). Temperature and pH were determined using a multiparameter (HI9829, Hanna Instruments, Woonsocket, RI, USA), whereas dissolved oxygen was measured using an oximeter (HI9147-04, Hanna Instruments, Woonsocket, RI, USA). Water hardness was determined using a LaMotte GH test kit (LaMotte Company, Chestertown, MD, USA), while total ammonia nitrogen, nitrite, and nitrate were quantified using JBL PRO AQUATEST (JBL GmbH & Co. KG, Neuhofen, Germany) colorimetric kits.

### 2.7. Chemical Analyses of Experimental Diets and Feces

Experimental diets from both trials and feces from the digestibility trial were analyzed according to the official methods of the Association of Official Analytical Chemists [21]. Moisture content was determined by oven drying. Crude protein was determined by the Kjeldahl method (N × 6.25). Crude lipid content was determined using ANKOM extraction equipment (ANKOM Technology, Macedon, NY, USA). Crude fiber was determined using the ANKOM Filter Bag Technique. Ash content was determined by incineration in a muffle furnace. Nitrogen-free extract (NFE) was estimated by difference. Gross energy was determined using a oxygen bomb calorimeter (Model 1341EE, Parr Instrument Company, Moline, IL, USA) following ASTM method D-2015-66 (1974) [22]. Chromium oxide III (Cr_2_O_3_) concentrations in diets and feces from the digestibility trial were analyzed separately for each replicate by atomic absorption spectrophotometry according to AOAC methods [25].

Chitin content in the experimental diets from the growth trial was determined spectrophotometrically following the procedure described by Eggink [26]. Briefly, samples were defatted with acetone, deproteinized with 0.5 M NaOH, hydrolyzed with 6 M HCl, and subjected to a colorimetric reaction. Absorbance was measured at 650 nm, and chitin content was calculated from the resulting spectrophotometric response.

### 2.8. Sampling and Serum Biochemistry

At the end of the growth trial, fish were fasted for 24 h. Subsequently, three fish from each tank (12 fish per treatment) were euthanized with eugenol (150 mg L^−1^). Blood samples were collected from the caudal vein using a 25-gauge needle attached to a 1 mL syringe and transferred to 1.5 mL serum separator tubes (Eppendorf SE, Hamburg, Germany) without anticoagulant. Samples were transported to an external laboratory for biochemical analysis.

Blood samples were centrifuged at 4500 rpm for 5 min within 30 min of collection (LLG uniCFUGE 5, LLG Labware, Meckenheim, Germany). Serum was recovered and 100 μL was dispensed into the sample tray of an automated biochemical analyzer (BK-200, Biobase Bioindustry Co., Ltd., Shandong, China). The following serum analytes were determined using commercial reagents (DiaSys Diagnostic Systems GmbH, Holzheim, Germany): glucose (GLU), total protein (TP), triglycerides (TG), cholesterol (CHOL), alanine aminotransferase (ALT), and aspartate aminotransferase (AST). The analyzer was calibrated using TruCal U calibration serum (DiaSys; lot 37463; exp. 29 August 2026).

### 2.9. Intestinal Histomorphology

Three fish per tank were sampled for histological evaluation of the distal intestine (DI). Approximately 1 cm of distal intestine was collected from each fish (n = 12 per treatment). Samples were washed with phosphate-buffered saline (PBS), gently blotted dry with absorbent paper, and fixed in Bouin’s solution for 24 h. Subsequently, samples were transferred to 70% ethanol until processing.

All samples were dehydrated through a graded ethanol series (50%, 60%, 70%, 85%, 90%, and 96%) using a tissue processor (HistoCore PEARL, Leica Biosystems, Nussloch, Germany) and embedded in paraffin using a paraffin dispenser (HistoCore Arcadia H, Leica Biosystems, Nussloch, Germany). Four cross-sections (4 μm thick) were obtained from each sample using a microtome (HistoCore AUTOCUT, Leica Biosystems, Nussloch, Germany), mounted on glass slides, and stained with hematoxylin and eosin using an Autostainer XL system (Leica Biosystems, Nussloch, Germany). All procedures, including tissue processing, embedding, and staining, were performed according to the methodology described by [27].

Digital images were captured using a Leica ICC50W digital camera (Leica Microsystems, Wetzlar, Germany) coupled to a Leica DM750 optical microscope (Leica Microsystems, Wetzlar, Germany) and analyzed using Leica Application Suite software (LAS v4.13.0, Leica Microsystems, Heerbrugg, Switzerland). Intestinal histomorphology was evaluated quantitatively. Six histomorphometric parameters were measured in each section: villus height (VL), villus width (VW), villus area (VA), goblet cell number (GC), muscularis thickness (MT), and mucosal thickness (M). Additional details of the measurements are presented in Section 3.

### 2.10. Statistical Analysis

Data are presented as mean ± standard deviation (SD). Statistical analyses were performed using SAS^®^ OnDemand for Academics (SAS ODA), version 9.4 (SAS Institute Inc., Cary, NC, USA). Data were analyzed using one-way analysis of variance (ANOVA) with a significance level of *p* < 0.05. The assumptions of normality and homogeneity of variances were assessed using the Shapiro–Wilk and Levene tests, respectively, whereas independence of errors was verified through examination of residual plots. When significant differences were detected, Tukey’s multiple comparison test was applied. When the assumptions of ANOVA were not met, the nonparametric Kruskal–Wallis test was used, followed by multiple comparisons using the Dwass–Steel–Critchlow–Fligner test.

## 3. Results

The results obtained in the digestibility trial are presented in (Table 5). High apparent digestibility coefficients (ADCs) were observed for the FF-BSFL meal, with values of 81.38% ± 3.58 for dry matter, 88.76% ± 2.14 for crude protein, 97.09% ± 1.13 for crude lipids, and 85.96% ± 2.94 for gross energy. These values corresponded to 38.39% ± 0.92 digestible protein, 28.3% ± 0.83 digestible lipids and 20.17 ± 0.16 MJ kg^−1^ digestible energy. Compared to fishmeal, the ADCs of the FF-BSFL meal were lower, although they remained high for all the nutrients evaluated.

All groups of fish accepted the experimental diets, and no mortality was recorded. For the growth trial, the growth performance and biometric indices of pacu are presented in (Table 6). Weight gain (WG), final biomass (FB), and feed intake (FI) were significantly affected by the fishmeal replacement levels with FF-BSFL (*p* < 0.001). Fish fed the HI30 diet showed no significant differences compared to the control diet (HI0), while those fed the HI60 and HI100 diets showed significantly lower values (*p* < 0.05). Similarly, the specific growth rate (SGR) did not differ between the HI30 and HI0 treatments, but was significantly lower in fish fed the HI60 and HI100 diets (*p* < 0.001).

Feed conversion ratio (FCR) showed significant differences between treatments (*p* = 0.019), with significantly higher values observed in fish fed the HI100 diet compared to those fed the HI0 diet, while the HI30 and HI60 diets showed intermediate values with no clear differences compared to the control. Condition factor (CF) also showed significant differences (*p* = 0.002), with the highest value recorded in fish fed the HI30 diet, which was significantly higher than that of those fed the HI60 and HI100 diets, but did not differ from that of fish fed the HI0 diet. On the other hand, protein efficiency ratio (PER) did not show significant differences between treatments (*p* = 0.058).

**Figure 1:**
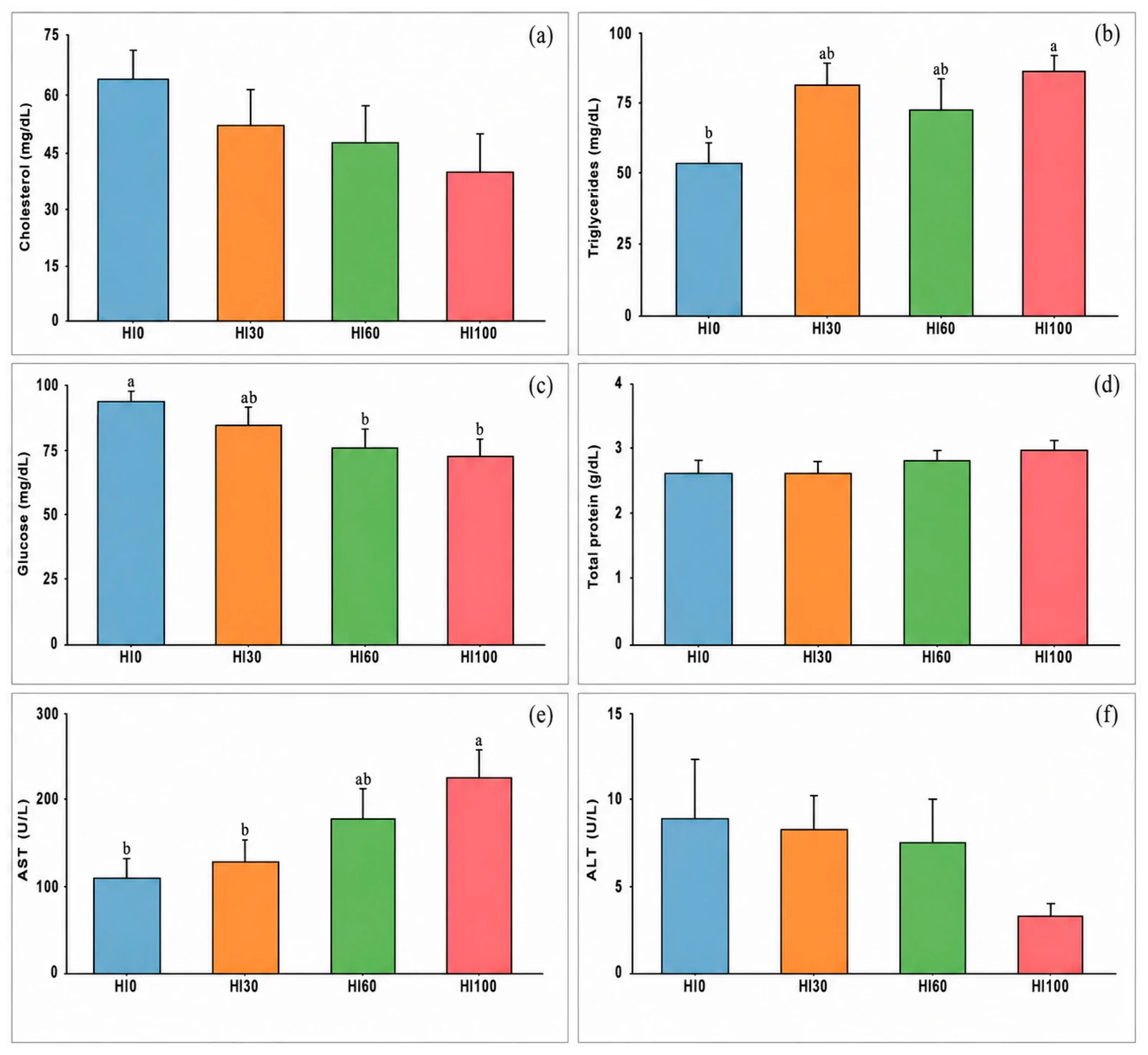
(**a**) Cholesterol (mg dL^−1^); (**b**) triglycerides (mg dL^−1^); (**c**) glucose (mg dL^−1^); (**d**) total protein (g dL^−1^); (**e**) AST (U L^−1^); and (**f**) ALT (U L^−1^). Different letters above the bars indicate significant differences between treatments. Data were analyzed using one-way ANOVA, after verifying the assumptions of normality (Shapiro–Wilk test) and homogeneity of variances (Levene’s test). When significant differences were detected (*p* < 0.05), Tukey’s multiple comparisons test was applied. Values are expressed as mean ± standard deviation.

Regarding serum biochemical parameters, shown in (Figure 1), cholesterol levels showed no significant differences between treatments (*p* > 0.05). Regarding triglycerides, significant differences were observed between treatments (*p* < 0.05), with higher values in fish fed without fishmeal (HI100) compared to the control diet (HI0), while fish fed HI30 and HI60 showed intermediate values. Blood glucose also showed significant differences (*p* < 0.05), with higher values recorded in fish fed the (HI0) diet compared to the treatments including insect meal, especially HI60 and HI100. Total protein levels showed no significant differences between treatments (*p* > 0.05). AST activity showed significant differences (*p* < 0.05), being higher in the blood of fish fed HI100 compared to the HI0 diet, with intermediate values in the HI30 and HI60 diets. ALT, on the other hand, showed no significant differences between treatments (*p* > 0.05).

The length, width, and area of the intestinal folds did not show significant differences between treatments (*p* > 0.05). In contrast, the number of goblet cells showed significant differences (*p* < 0.001), with higher values in HI0 and HI30 compared to HI60 and HI100. Similarly, the thickness of the mucosa and muscularis layer also showed significant differences (*p* < 0.001), with higher values in HI0 and HI30 and lower values in HI60 and HI100. The histomorphological indices of the distal intestine of pacu are presented in (Table 7).

**Figure 2:**
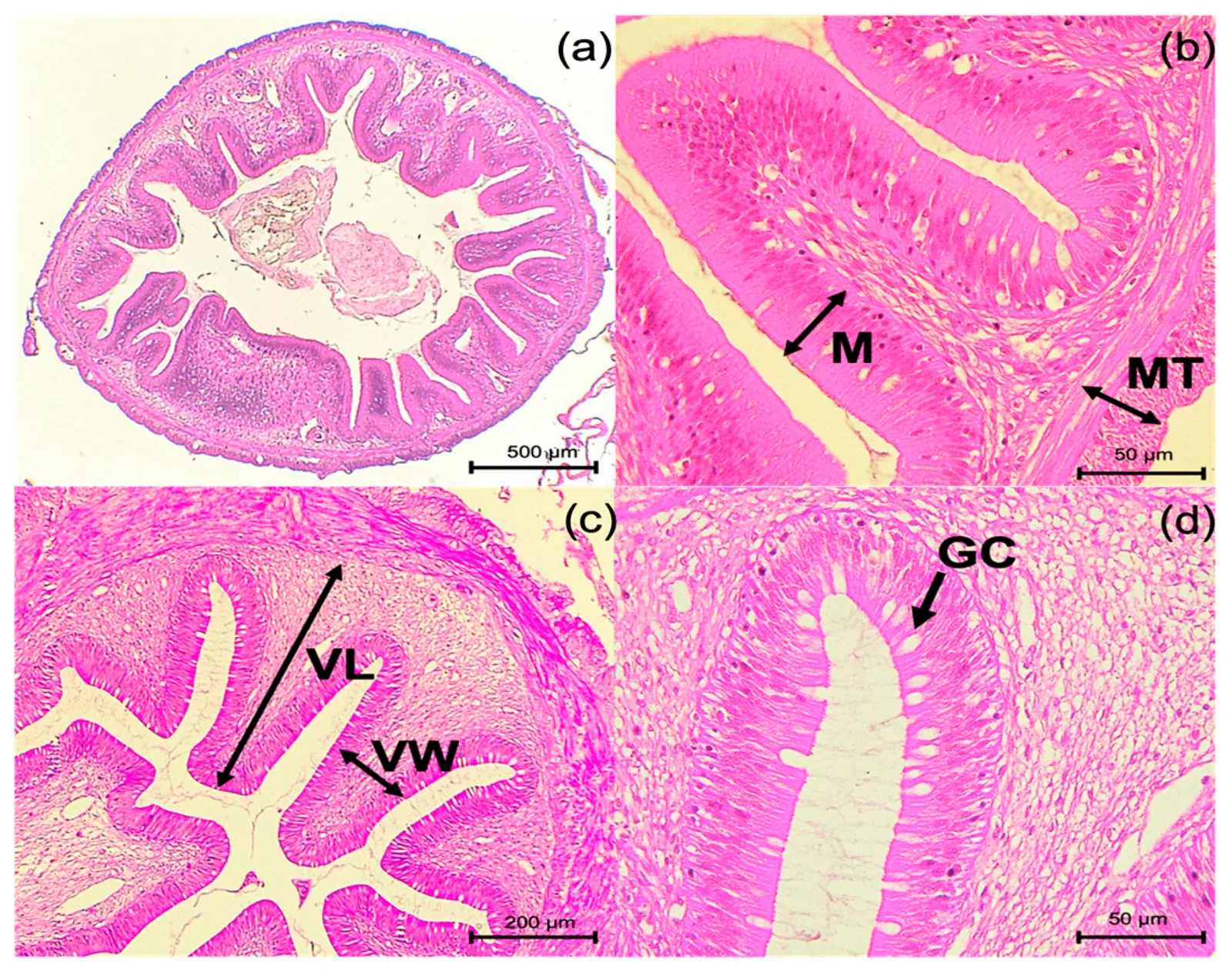
Histomorphology of the distal section of the intestine of the pacu (*Piaractus brachypomus*). (**a**) General view of the distal intestine showing the organization of the mucosa and intestinal villus (bar = 500 µm). (**b**) Arrows show how the mucosal thickness (M) and muscularis thickness (MT) were measured (bar = 50 µm). (**c**) Arrows show how the length (VL) and width (VW) of the intestinal villus were measured (bar = 200 µm). (**d**) Number of goblet cells (GC) in the epithelium of the distal section of the intestine (bar = 50 µm).

The histomorphological characteristics of the distal intestine of the pacu revealed the organization of the mucosa, intestinal folds, and the presence of goblet cells in the epithelium. These characteristics are illustrated in (Figure 2).

## 4. Discussion

The evaluation of alternative ingredients in aquaculture typically considers not only their nutritional composition but also their effect on growth, feed efficiency, and the physiological state of farmed organisms, which are commonly used to determine their suitability in formulated diets [28]. The proximate composition of the FF-BSFL meal used in this study fell within the ranges reported in the literature for this type of ingredient, which is characterized by its high lipid and protein content, associated with a high energy value [8,29]. These values can be influenced by the nutritional composition of the substrate supplied to the larvae, as this factor directly conditions nutrient accumulation and, consequently, the variability in the chemical composition of the insect meal [8].

This study represents the first report on the digestibility of BSFL meal in pacu (*Piaractus brachypomus*). The results showed high apparent digestibility coefficients (ADCs) for crude protein (88.76%), lipids (97.09%), and gross energy (85.96%). While these values were lower than those observed for fishmeal, they remained within an acceptable range, maintaining the potential of this ingredient for use in aquaculture formulations. Along these lines, it has been reported that the digestibility of BSFL meal can vary depending on its compositional and structural characteristics, although it generally remains at high levels [30].

Similarly, in tambaqui (*Colossoma macropomum*), diets with FF-BSFL achieved CDAs exceeding 88% for protein and energy, and 96.42% for lipids [16]. Taken together, these findings reinforce the potential of this ingredient as a highly usable source for omnivorous Neotropical characins [20], which could be related to the fact that insects are part of the natural diet of these species and that their gastrointestinal tract possesses chitinase to facilitate the digestion of chitin from insects and crustaceans [10].

A lipid apparent digestion rate (ADR) exceeding 97% in pacu demonstrates a high capacity for utilizing the lipid fraction of FF-BSFL, placing it in the upper range of values reported for insect-derived ingredients [10]. In rainbow trout fed DF-BSFL, for example, values close to 73% for lipids and 75% for dry matter have been reported [31], which could suggest greater efficiency of pacu in lipid digestion. This difference could be associated with both species-specific characteristics and the lipid profile of BSFL, which has been highlighted as a relevant energy source in aquaculture diets [32,33].

The digestibility of the dry matter (81.38%), on the other hand, was lower than that observed for lipids, suggesting less utilization of the ingredient, although still within high ranges. This behavior would indicate that a fraction of the FF-BSFL was not completely digested, which may be associated with the presence of less digestible structural components of the insect exoskeleton, such as chitin. This compound has been identified as one of several factors that may contribute to lower nutrient digestibility in fish due to its limited degradation in the digestive tract [26,34].

Furthermore, due to the non-protein nitrogen (NPN) content present in chitin, several authors have recommended using nitrogen conversion factors lower than 6.25 to more accurately estimate the crude protein content in insect-derived meals [35]. However, due to the lack of a comprehensive and standardized database for these ingredients, the conventional factor N × 6.25 was used in this study, a criterion also employed in previous studies with insect meals [36,37]. Although some authors recommend applying corrections related to the chitin fraction [18,38], there is currently no methodological consensus on the most appropriate nitrogen-to-protein conversion factor for this type of ingredient. Furthermore, applying these factors to correct apparent digestibility would involve estimating a new coefficient based on the estimated non-protein nitrogen present in feces, which is presumably much more abundant than in feed.

The growth performance results indicated that replacing fishmeal with FF-BSFL at levels up to 30% did not compromise the growth of juvenile pacu. However, higher replacement levels (HI60 and HI100) reduced growth performance. The lower growth observed at these replacement levels appeared to be primarily associated with reduced feed intake, which decreased by approximately 35% as the replacement level increased. Previous studies have reported that increasing dietary levels of BSFL meal may reduce feed intake due to lower palatability, a greater satiating effect, or the presence of chitin [16,18,26].

However, the reduced growth performance observed at the highest fishmeal replacement levels should not be interpreted solely as a direct effect of replacing fishmeal with FF-BSFL. In the present study, increasing FF-BSFL replacement while maintaining isoenergetic and isoproteic diets was associated with changes in the dietary matrix, including higher soybean meal and FF-BSFL levels, lower corn meal levels, reduced calculated n-3 fatty acids, and higher crude lipid and crude fiber contents. Although the diets were formulated to be isoenergetic, HI100 showed slightly higher analyzed gross energy and crude lipid content. Previous studies have shown that increasing dietary *H. illucens* meal can modify lipid quality and fatty acid composition [39], while feed utilization may also be affected by dietary lipid content and fishmeal replacement level [18,40]. Therefore, the lower growth observed at the highest replacement levels likely reflects the combined effects of FF-BSFL, associated changes in diet composition, and reduced feed intake.

Regarding serum biochemical parameters, cholesterol concentrations were not significantly affected by dietary treatment (*p* > 0.05), although a decreasing trend was observed with increasing levels of fishmeal replacement by FF-BSFL. These findings suggest that juvenile pacu maintained lipid homeostasis despite the replacement of fishmeal with insect meal. Similar responses have been reported in gilthead seabream and Atlantic salmon fed diets containing black soldier fly larvae meal [41,42].

Conversely, triglycerides increased significantly in the total replacement group, while glucose decreased. The combination of these responses may suggest changes in energy metabolism, with a possible greater involvement of lipid-derived energy in fish fed high-FF-BSFL diets, as also reported in African catfish and carp [43,44,45]. This interpretation is supported by the high lipid digestibility observed for FF-BSFL (ADC > 97%), suggesting that pacu was able to efficiently utilize the lipid fraction of this ingredient.

However, because metabolic enzyme activities, liver and muscle glycogen, and lipid deposition were not evaluated, this mechanism cannot be confirmed. Therefore, the changes in serum triglycerides and glucose concentrations may also reflect reduced feed intake, handling-related stress, altered lipid absorption or transport, and broader changes in dietary composition, including the higher crude lipid content and lower calculated n-3 fatty acid levels observed in the HI100 diet.

AST activity increased significantly at the highest replacement level, whereas ALT levels remained stable (*p* > 0.05). Since ALT is considered a more liver-associated indicator in fish, its stability may suggest the absence of evident hepatocellular alteration [46,47]. However, increased AST activity should not be considered a specific indicator of hepatic damage in fish, because this enzyme is also present in extrahepatic tissues, such as muscle [48]. Thus, the increase in AST observed in HI100 should be considered a nonspecific physiological response, possibly associated with greater metabolic demand, dietary changes, or handling-related stress, rather than direct evidence of liver damage [45]. Additional hepatic, oxidative, or histological indicators would be required to more precisely assess liver status.

Replacement of the fishmeal up to 100% (HI100) did not significantly affect the length, width, or area of the distal intestinal folds. This preservation of the absorptive architecture is consistent with previous research in rainbow trout and Siberian sturgeon, where moderate to high levels of insect meal inclusion also did not compromise the basic structural integrity of the intestinal folds [49,50].

However, in this study, high replacement levels (HI60 and HI100) were associated with a marked and significant reduction in mucosal thickness and muscularis thickness. Similar structural thinning has been documented in Jian carp, where fishmeal replacements exceeding 50% with *H. illucens* significantly reduced columnar epithelial and muscle layer thickness [51]. In the present study, dietary chitin increased progressively with increasing fishmeal replacement by FF-BSFL, from 0% in HI0 to 3.10% in HI100, while analyzed crude lipid content remained between 4.02% and 4.46% in HI0–HI60 and increased to 6.94% in the total replacement diet.

These changes, together with the increase in crude fiber and broader modifications in overall dietary composition, suggest that the intestinal alterations observed at high replacement levels were likely associated with combined dietary effects rather than with a single dietary factor. This interpretation is consistent with recent reviews indicating that fishmeal replacement levels above 50% with FF-BSFL may adversely affect intestinal morphology, partly due to the high lipid and chitin fractions of FF-BSFL [52].

Furthermore, the significant decrease in the number of goblet cells in the higher replacement treatments may suggest a potential impairment of the intestinal barrier’s protective function. This result contrasts with what has been reported in species such as rainbow trout, where the presence of dietary chitin stimulated a compensatory overproduction of mucins and an increase in goblet cells, promoting epithelial lubrication [53]. The slight decrease in the number of these secretory cells in pacu may indicate intestinal physiological stress, potentially associated with morphometric alterations, epithelial detachment, and signs of enteritis described in gilthead seabream and hybrid grouper under high insect meal inclusions [54,55].

In this sense, these histological alterations may help to explain the lower efficiency in nutrient utilization and the reduction in growth observed in this study at replacement levels above 30%, which limits the viability of FF-BSFL as a total replacement in this species [51,52].

However, the changes observed in the intestine could also be explained as a consequence of a significant decrease in intake, so a greater substitution could be possible by partially defatting or improving palatability.

## 5. Conclusions

Full-fat black soldier fly meal showed high digestibility coefficients in pacu. Replacing up to 30% of the fishmeal allowed for adequate growth without affecting biochemical parameters or intestinal integrity. However, higher levels compromised growth performance and caused physiological and intestinal alterations. Therefore, under the conditions of this study, a 30% replacement of fishmeal by FF-BSFL is recommended for juvenile pacu diets.

## Figures and Tables

**Table 1:** Formulation and nutritional composition of the reference diet utilized in the digestibility trial.

Ingredients	Reference Diet (%)
Soybean meal	39.80
Rice meal	25.87
Prime fishmeal	19.90
Corn meal	7.96
Soybean oil	4.98
Calcium carbonate	0.58
Aquaculture premix ^1^	0.20
Salt	0.11
Choline chloride	0.10
Chromium oxide III	0.50
Total	100
Nutritional composition	
Dry matter (%)	92.11
Crude protein (%)	34.28
Crude lipids (%)	5.29
Crude fiber (%)	2.18
Ashes (%)	7.07
Gross energy (MJ kg^−1^)	18.12

^1^ Vitamins (mg kg^−1^ diet). A: 9334 IU; D3: 1867 IU; E: 93 IU; K3: 5; B1: 12; B2: 13; B6: 10; B12: 0.02; Ascorbic acid: 210; Niacin: 100; Pantothenic Acid: 33; Folic Acid: 2.67; Biotin: 0.27. Minerals (mg kg^−1^ diet). Manganese: 26.6; Iodine: 1; Copper: 1; Zinc: 13.3; Iron: 13.3; Cobalt: 0.10; Selenium: 0.20.

**Table 2:** Ingredient formulation and calculated nutritional composition of the experimental diets (%, dry matter basis).

	Experimental Diets			
Ingredients (%)	HI0	HI30	HI60	HI100
Prime fishmeal, 65%	30.00	21.00	12.00	-
Soybean meal, 47%	26.00	31.00	36.00	45.00
Corn meal	21.43	17.28	12.98	4.15
Broken rice	20.00	20.00	20.00	20.00
Full-Fat BSF meal, 43%	-	9.00	18.00	30.00
Soybean oil	2.00	1.10	0.30	0.00
Aquaculture premix ^1^	0.20	0.20	0.20	0.20
Salt	0.12	0.12	0.12	0.12
DL-Methionine	-	0.05	0.15	0.28
Fungal inhibitor ^2^	0.15	0.15	0.15	0.15
Mycotoxin binder ^3^	0.06	0.06	0.06	0.06
Antioxidant ^4^	0.04	0.04	0.04	0.04
	Experimental Diets			
Calculated nutritional composition	HI0	HI30	HI60	HI100
Dry matter	90.09	90.46	90.89	91.72
Crude protein	35.20	34.67	34.18	34.00
Crude lipids	6.11	7.29	8.59	10.94
Crude fiber	1.62	2.14	2.66	3.31
Digestible energy (MJ kg^−1^)	14.23	14.23	14.23	14.23
Lysine	2.45	2.36	2.28	2.26
Methionine	0.86	0.84	0.92	1.03
Arginine	2.32	2.32	2.32	2.36
Threonine	1.47	1.45	1.44	1.43
Tryptophan	0.45	0.47	0.48	0.52
Valine	1.92	1.97	2.02	2.10
Methionine + cystine	1.30	1.20	1.20	1.20
Phenylalanine + tyrosine	2.95	2.68	2.40	2.07
Calcium	1.20	1.04	1.00	1.38
Phosphorus	1.15	1.02	0.90	0.80
omega-3 fatty acids	1.38	1.11	0.84	0.54
omega-6 fatty acids	1.79	2.00	2.27	2.96

^1^ Vitamins (mg kg^−1^ diet). A: 9334 IU; D3: 1867 IU; E: 93 IU; K3: 5; B1: 12; B2: 13; B6: 10; B12: 0.02; Ascorbic acid: 210; Niacin: 100; Pantothenic Acid: 33; Folic Acid: 2.67; Biotin: 0.27. Minerals (mg kg^−1^ diet). Manganese: 26.6; Iodine: 1; Copper: 1; Zinc: 13.3; Iron: 13.3; Cobalt: 0.10; Selenium: 0.20. ^2^ Novamold 0.10% and Alquermold 0.05%. ^3^ Alquerfeed 0.06%. ^4^ Antioxidant: Butylated hydroxytoluene (BHT).

**Table 3:** Analyzed chemical composition and chitin of the experimental diets (%, dry matter basis).

	Experimental Diets			
Nutrients (%)	HI0	HI30	HI60	HI100
Dry matter	90.28	91.38	91.69	93.10
Crude protein	34.81	34.35	34.13	34.11
Crude lipids	4.24	4.02	4.46	6.94
Crude fiber	1.46	2.07	2.78	4.03
Chitin	0	1.14	2.08	3.10
Ashes	7.16	6.79	6.83	6.80
Gross energy (MJ kg^−1^)	17.20	17.28	17.28	17.87

**Table 4:** Water quality parameters during the experimental period of the digestibility and growth trial of pacu.

		Digestibility Trial		Growth Trial	
Parameters	Time	Mean	SD	Mean	SD
Aquarium temperature (°C)	8:00	28.92	±1.05	28.23	±0.47
	12:00	29.98	±0.72	28.76	±0.48
	17:00	30.16	±0.39	28.35	±0.48
Dissolved oxygen (mg L^−1^)		6.08	±0.30	5.20	±0.09
pH		6.61	±0.28	7.29	±0.12
Ammonia (mg L^−1^)		0.47	±0.33	0.26	±0.01
Nitrite (mg L^−1^)		0.32	±0.11	0.20	±0.01
Nitrate (mg L^−1^)		15.00	±5.16	31.15	±0.78
Water hardness (mg L^−1^)		36.63	±16.60	23.51	±0.73

Values are presented as mean ± standard deviation (SD).

**Table 5:** Chemical composition, apparent digestibility coefficients (ADCs), and digestible nutrient and energy values of full-fat black soldier fly larvae meal (FF-BSFL) and fishmeal (FM), calculated using the test diets from the digestibility trial.

Nutrient (%) and Energy (MJ kg^−1^)	FF-BSFL (Mean ± SD)	FM (Mean ± SD)
Dry matter	96.12	92.80
Crude protein	43.25	65.25
Crude lipids	29.15	10.33
Gross energy	23.44	19.32
Apparent Digestibility Coefficient (%)		
Dry matter	81.38 ± 3.58	86.13 ± 2.13
Dry matter	81.38 ± 3.58	86.13 ± 2.13
Crude protein	88.76 ± 2.14	94.35 ± 0.98
Crude lipids	97.09 ± 1.13	99.42 ± 0.31
Gross energy	85.96 ± 2.94	89.40 ± 2.24
**Digestible Nutrients and Energy of FF-BSFL**		
Dry matter (%)	78.32 ± 3.44	79.92 ± 1.97
Digestible protein (%)	38.39 ± 0.92	61.56 ± 0.64
Digestible lipids (%)	28.30 ± 0.83	10.27 ± 0.03
Digestible energy (MJ kg^−1^)	20.17 ± 0.16	18.49 ± 0.44

**Table 6:** Growth performance and biometric indices of pacu fed diets containing graded levels of FF-BSFL meal for 60 days.

Diets	IW	WG	FB	FI	FCR	SGR	CF	PER
HI0	3.25 ± 0.11	47.55 ± 2.55 ^a^	1003.88 ± 67.99 ^a^	1166.60 ± 49.63 ^a^	1.24 ± 0.05 ^a^	4.58 ± 0.12 ^a^	1.78 ± 0.01 ^ab^	2.31 ± 0.09
HI30	3.22 ± 0.07	43.88 ± 1.72 ^a^	941.50 ± 33.40 ^a^	1119.83 ± 18.68 ^a^	1.28 ± 0.05 ^ab^	4.48 ± 0.09 ^a^	1.84 ± 0.03 ^a^	2.28 ± 0.09
HI60	3.20 ± 0.03	28.33 ± 1.42 ^b^	630.50 ± 28.49 ^b^	740.88 ± 41.73 ^b^	1.31 ± 0.03 ^ab^	3.81 ± 0.08 ^b^	1.71 ± 0.03 ^b^	2.24 ± 0.05
HI100	3.28 ± 0.06	27.28 ± 1.45 ^b^	611.00 ± 28.87 ^b^	738.30 ± 31.86 ^b^	1.35 ± 0.03 ^b^	3.72 ± 0.09 ^b^	1.76 ± 0.05 ^b^	2.17 ± 0.05
*p*-value	0.574	<0.001	<0.001	<0.001	0.019	<0.001	0.002	0.058

IW: initial weight; WG: weight gain; FB: final biomass; FI: feed intake; FCR: feed conversion ratio; SGR: specific growth rate; CF: condition factor; PER: protein efficiency ratio. Different letters in the same column indicate significant differences between treatments. Data were analyzed using one-way ANOVA, after verifying the assumptions of normality (Shapiro–Wilk test) and homogeneity of variances (Levene’s test). When significant differences were detected (*p* < 0.05), Tukey’s multiple comparisons test was applied.

**Table 7:** Histomorphological indices of the distal intestine of pacu (*Piaractus brachypomus*) fed diets with increasing levels of FF-BSFL.

Treatment	Villus Length (µm)	Villus Width (µm)	Villus Area (µm^2^)	Number of Goblet Cells	Mucosal Thickness (µm)	Muscularis Thickness (µm)
HI0	432.2 ± 53.4	179.8 ± 19.7	70,685 ± 5564	58.06 ± 3.8 ^a^	51.80 ± 0.2 ^a^	96.45 ± 5.7 ^a^
HI30	362.8 ± 75.7	168.7 ± 13.6	58,032 ± 12,923	57.22 ± 4.4 ^a^	50.09 ± 1.2 ^a^	94.25 ± 7.5 ^a^
HI60	401.8 ± 65.3	165.9 ± 7.9	62,856 ± 14,052	45.15 ± 3.8 ^b^	43.12 ± 4.0 ^b^	76.02 ± 7.7 ^b^
HI100	388.6 ± 26.9	154.0 ± 3.4	56,429 ± 2917	45.75 ± 2.5 ^b^	42.96 ± 2.0 ^b^	61.94 ± 5.8 ^b^
*p*-value	0.438 *	0.057 **	0.210 **	<0.001 *	<0.001 *	<0.001 *

Different letters in the same column indicate significant differences between treatments. Data are expressed as mean ± standard deviation. (*) Data were analyzed using one-way ANOVA, after verifying the assumptions of normality (Shapiro–Wilk test) and homogeneity of variances (Levene’s test). When significant differences were detected (*p* < 0.05), Tukey’s multiple comparisons test was applied. (**) When data did not meet the assumptions of normality or showed heterogeneity of variances, the non-parametric Kruskal–Wallis test was used, followed by multiple comparisons using the Dwass–Steel–Critchlow–Fligner test.

## Data Availability

The original data supporting the findings of this study are available in the Supplementary Materials. Further inquiries can be directed to the corresponding author.

## Supplementary Materials

The following supporting information can be downloaded at: https://www.mdpi.com/article/10.3390/ani16172741/s1, Table S1. Nutritional composition of the full-fat black soldier fly larvae (*Hermetia illucens*) meal; Table S2. Heavy metal concentrations and microbiological analysis of the full-fat black soldier fly larvae meal; Table S3. Chitin content of the experimental diets used in the growth trial; Table S4. Proximate composition of the experimental diets used in the digestibility trial; Table S5. Proximate composition of the feces collected during the digestibility trial; Table S6. Chromium (III) oxide (Cr_2_O_3_) content in the experimental diets used in the digestibility trial; Table S7. Chromium(III) oxide (Cr_2_O_3_) content in feces from the digestibility trial; Table S8. Growth performance of pacu fed the experimental diets used in the growth trial; Table S9. Blood biochemical parameters of pacu fed the experimental diets used in the growth trial; Table S10. Morphometric indices of the distal intestine of pacu fed the experimental diets used in the growth trial.
