# Supplementary material for: Full-Fat Black Soldier Fly Larvae Meal in Diets for Pacu, Piaractus brachypomus: Digestibility, Growth Performance, Blood Biochemistry and Gut Morphology
Source: Animals (Basel). 2026 Sep 2;16(17):2741. doi: 10.3390/ani16172741 (PMC13565431; doi:10.3390/ani16172741)
Supplement: Supplementary file 1 [file animals-16-02741-s001.zip › animals-4391563-supplementary.pdf]

FULL-FAT BLACK SOLDIER FLY LARVAE MEAL IN DIETS FOR PACU, *Piaractus brachypomus*: DIGESTIBILITY, GROWTH PERFORMANCE, BLOOD BIOCHEMISTRY AND GUT MORPHOLOGY

Table S1. Nutritional composition of the full-fat black soldier fly larvae (*Hermetia illucens*) meal.

Full-fat black soldier fly larvae meal			
**Proximate composition (%)**		**Fatty acids (% of total fatty acids)**	
Crude protein	43.25	**Saturated**	
Ether extract	29.15	Lauric acid	31.183
Crude fiber	6.99	Myristic acid	6.579
Ash	11.26	Palmitic acid	16.411
Nitrogen-free extract	5.47	Stearic acid	3.835
Gross energy (MJ/kg)	23.44	Heneicosanoic acid	1.068
**Amino acids (g/100 g)**		Tricosanoic acid	0.778
Serine	1.2	**Monounsaturated**	
Arginine	2.9	cis-9 Oleic acid	23.098
Glycine	2.6	Palmitoleic acid	2.689
Aspartic acid	1.9	Myristoleic acid	0.166
Glutamic acid	4.4	**Polyunsaturated**	
Threonine	0.9	Linoleic acid (Omega-6)	13.159
Alanine	2.3	Arachidic acid (Omega-6)	0.124
Proline	2.1	Linolenic acid (Omega-3)	0.592
Cysteine	0.1	EPA (Omega-3)	0.165
Lysine	2.4		
Tyrosine	2.5		
Methionine	0.5		
Valine	2.5		
Isoleucine	1.8		
Leucine	3.1		
Phenylalanine	1.9		
Tryptophan	1.0		

Table S2. Heavy metal concentrations and microbiological analysis of the full-fat black soldier fly larvae meal.

**Metal**	**Result (mg/kg)**
Arsenic (As)	<0.06
Cadmium (Cd)	<0.05
Lead (Pb)	<0.1
Mercury (Hg)	<0.05
Chromium (Cr)	<0.1
Nickel (Ni)	<0.1
Copper (Cu)	7.28
Zinc (Zn)	57.2
Iron (Fe)	212
Selenium (Se)	<0.25
Manganese (Mn)	37.7
**Microbiological analysis**	
Salmonella	Absence
E. coli (CFU/g)	<10

CFU, colony-forming units.

Table S3. Chitin content of the experimental diets used in the growth trial.

Identification	Assay	Unit	Result
HI0	Chitin	%	0.00
HI30	Chitin	%	1.14
HI60	Chitin	%	2.08
HI100	Chitin	%	3.10

**The results are expressed as percentage of chitin (g of chitin / 100 g of sample).*

Table S4. Proximate composition of the experimental diets used in the digestibility trial.

Diet	Moisture (%)	Crude protein (N × 6.25) (%)	Ether extract (%)	Gross energy (MJ/kg)	Crude fiber (%)
Reference diet (T1)	7.89	34.28	5.29	18.113	2.18
Fishmeal diet (T2)	7.66	43.42	8.27	18.414	1.89
FF-BSFL diet (T3)	6.56	34.70	13.55	19.669	3.00

FF-BSFL, full-fat black soldier fly larvae meal.

Table S5. Proximate composition of the feces collected during the digestibility trial.

Sample	Moisture (%)	Protein (%)	Ether extract (%)	Energy by calorimetry (MJ/kg)	Crude fiber (%)
T1R1	10.39	13.30	0.3	14.38	23.23
T1R2	10.44	12.43	0.4	14.03	22.00
T1R3	10.48	12.94	0.68	14.42	24.03
T2R1	11.54	16.07	0.44	13.09	17.67
T2R2	10.41	16.55	0.28	13.38	18.95
T2R3	10.12	16.78	0.21	12.64	17.22
T3R1	9.52	20.37	1.5	14.05	25.97
T3R2	10.69	19.94	2.12	14.18	27.49
T3R3	9.59	20.25	2.34	14.55	25.65

T, treatment; R, replicate.

Table S6. Chromium (III) oxide (Cr_2O_3) content in the experimental diets used in the digestibility trial.

Diet	Cr_2O_3 (%)
Reference diet (T1)	0.57
Fishmeal diet (T2)	0.54
FF-BSFL diet (T3)	0.47

FF-BSFL, full-fat black soldier fly larvae meal.

Table S7. Chromium(III) oxide (Cr_2O_3) content in feces from the digestibility trial.

Sample	Cr_2O_3 (%)
T1R1	3.19
T1R2	3.11
T1R3	3.59
T2R1	3.12
T2R2	3.43
T2R3	4.6
T3R1	3.05
T3R2	2.38
T3R3	2.06

T, treatment; R, replicate.

Table S8. Growth performance of pacu fed the experimental diets used in the growth

Treatment	IW (g)	FW (g)	WG trial (g)	Feed intake (g)	FCR	SGR	PER	CF
1	3.4	48.5	45.1	1099.2	1.29	4.43	2.23	1.78
1	3.3	54.1	50.8	1199.3	1.18	4.66	2.43	1.80
1	3.2	49.2	46	1159.7	1.26	4.55	2.28	1.77
1	3.1	51.4	48.3	1208.2	1.25	4.68	2.30	1.78
2	3.1	47.2	44.1	1092.5	1.24	4.54	2.35	1.82
2	3.3	44.7	41.4	1123.1	1.36	4.34	2.15	1.84
2	3.2	48.5	45.3	1131.7	1.25	4.53	2.33	1.81
2	3.2	47.9	44.7	1132	1.27	4.51	2.30	1.88
3	3.2	31.6	28.4	757.6	1.33	3.82	2.20	1.68
3	3.2	32.3	29.1	772.3	1.33	3.85	2.21	1.71
3	3.2	32.7	29.5	754.2	1.28	3.87	2.29	1.74
3	3.2	29.5	26.3	679.4	1.29	3.70	2.27	1.73
4	3.2	32.3	29.1	774.1	1.33	3.85	2.20	1.72
4	3.2	28.8	25.6	698.3	1.37	3.66	2.15	1.78
4	3.3	30.8	27.5	731.6	1.33	3.72	2.20	1.72
4	3.4	30.3	26.9	749.2	1.39	3.65	2.11	1.82

Treatment, dietary group (1–4). IW, initial weight; FW, final weight; WG, weight gain; FCR, feed conversion ratio; SGR, specific growth rate; PER, protein efficiency ratio; CF, condition factor.

Table S9. Blood biochemical parameters of pacu fed the experimental diets used in the growth trial.

Treatment	Triglycerides (mg/dL)	Cholesterol (mg/dL)	ALT (U/L)	AST (U/L)	Glucose (mg/dL)	Total protein (g/dL)
1	69.57	46.74	15.65	104.68	91.89	2.36
1	51.16	59.55	8.68	106.32	97.67	3.06
1	41.61	55.09	6.97	102.23	96.00	2.73
1	54.95	91.88	6.71	123.31	91.17	3.02
2	87.32	65.63	9.90	95.50	88.83	2.49
2	70.06	30.51	7.89	113.44	79.00	2.67
2	71.75	40.90	8.91	148.71	92.50	2.95
2	96.64	66.24	9.28	161.86	79.94	3.08
3	90.85	70.33	8.08	156.89	76.89	3.40
3	79.03	67.81	11.68	128.52	73.89	3.15
3	79.84	31.03	2.39	158.46	68.78	2.85
3	42.17	16.02	8.46	231.34	80.83	2.43
4	91.50	24.49	3.37	227.20	70.00	3.06
4	85.81	57.07	3.28	207.67	77.50	3.10
4	89.52	49.98	5.39	148.30	64.67	3.03
4	75.67	25.77	3.44	325.44	80.67	3.10

Treatment, dietary group (1–4). ALT, alanine aminotransferase; AST, aspartate aminotransferase.

Table S10. Morphometric indices of the distal intestine of pacu fed the experimental diets used in the growth trial.

Treatment	Villus width (μm)	Villus length (μm)	Villus area (μm²)	Number of goblet cells	Mucosal thickness (μm)	Muscularis thickness (μm)
1	443	161	64549.33	58.03	51.58	99.22
1	503	164	78021.67	62.40	51.99	100.03
1	396	195	69498.67	58.73	51.94	98.61
1	386	199	70668.33	53.07	51.68	87.96
2	322	175	54284.33	61.47	48.36	101.04
2	470	161	73213.67	60.87	50.24	99.60
2	299	154	42540.00	49.40	51.19	85.09
2	361	185	62089.00	57.13	50.57	91.26
3	364	166	56428.00	50.20	42.80	80.08
3	360	165	54908.00	46.20	41.41	81.17
3	498	176	83909.67	41.07	41.16	64.55
3	385	157	56178.33	43.13	47.11	78.29
4	410	152	56246.67	48.87	45.40	69.91
4	378	159	57203.33	45.20	40.96	61.20
4	356	151	52618.00	42.87	43.77	56.05
4	411	154	59648.67	46.07	41.73	60.61

Treatment, dietary group (1–4).
